# Three-Dimensional Modeling of Glucose-6-phosphate Dehydrogenase-Deficient Variants from German Ancestry

**DOI:** 10.1371/journal.pone.0000625

**Published:** 2007-07-18

**Authors:** Farooq Kiani, Sonja Schwarzl, Stefan Fischer, Thomas Efferth

**Affiliations:** 1 Computational Biochemistry, Interdisciplinary Center for Biocomputing, University of Heidelberg, Heidelberg, Germany; 2 Pharmaceutical Biology, German Cancer Research Center, Heidelberg, Germany; Temasek Life Sciences Laboratory, Singapore

## Abstract

**Background:**

Loss of function of dimeric glucose-6-phosphate dehydrogenase (G6PD) represents the most common inborn error of metabolism throughout the world affecting an estimated 400 million people. In Germany, this enzymopathy is very rare.

**Methodology/Principal Findings:**

On the basis of G6PD crystal structures, we have analyzed six G6PD variants of German ancestry by three-dimensional modeling. All mutations present in the German population are either close to one of the three G6P or NADP^+^ units or to the interface of the two monomers. Two of the three mutated amino acids of G6PD Vancouver are closer to the binding site of NADP^+^. The G6PD Aachen mutation is also closer to the second NADP^+^ unit. The G6PD Wayne mutation is closer to the G6P binding region. These mutations may affect the binding of G6P and NADP^+^ units. Three mutations, i.e. G6PD Munich, G6PD Riverside and G6PD Gastonia, lie closer to the interface of the two monomers. These may also affect the interface of two monomers.

**Conclusion:**

None of these G6PD variants share mutations with the common G6PD variants known from the Mediterranean, Near East, or Africa indicating that they have developed independently. The G6PD variants have been compared with mutants from other populations and the implications for survival of G6PD variants from natural selection have been discussed.

## Introduction

Loss of function of dimeric glucose-6-phosphate dehydrogenase (G6PD) represents the most common inborn error of metabolism throughout the world affecting an estimated 400 million people [1]. Prolonged neonatal jaundice and hemolytic anemia are common clinical manifestations. Infections, ingestion of fava beans, and some drugs can trigger life-threatening hemolytic anemia. G6PD is the first enzyme of the pentose phosphate pathway that converts β-D-glucose-6-phosphate into D-glucono-1,5-lactone-6-phosphate and is involved in the generation of NADPH [2]. As erythrocytes lack the citric acid cycle, the pentose phosphate shunt is the only source of NADPH. NADPH is required for the generation of reduced glutathione, which is important for the protection against oxidative damage.

As the G6PD gene is located at the X-chromosome at Xq28 [3] the disease is recessively inherited in males. In the past, more than 400 variants have been proposed based on clinical and enzymatic properties [4]. Precise molecular characterization of the G6PD gene showed that these 400 variants correspond to only 140 mutations. These are mainly 140 missense mutations leading to amino-acid substitutions and in a few cases base pair deletions that do not produce frame shifts are known [5]. Few splicing mutations have been documented [6], [7]. American, Mediterranean, and African ancestries are the best analyzed forms as of yet. In contrast to Southern European countries, only few variants are known from Middle and Northern Europe [8], [9].

In extension to previous investigations on the three-dimensional structure of G6PD and structural localization of variants [10], [11], we now report the modeling of G6PD variants of German origin.

## Results

**Figure 1:**
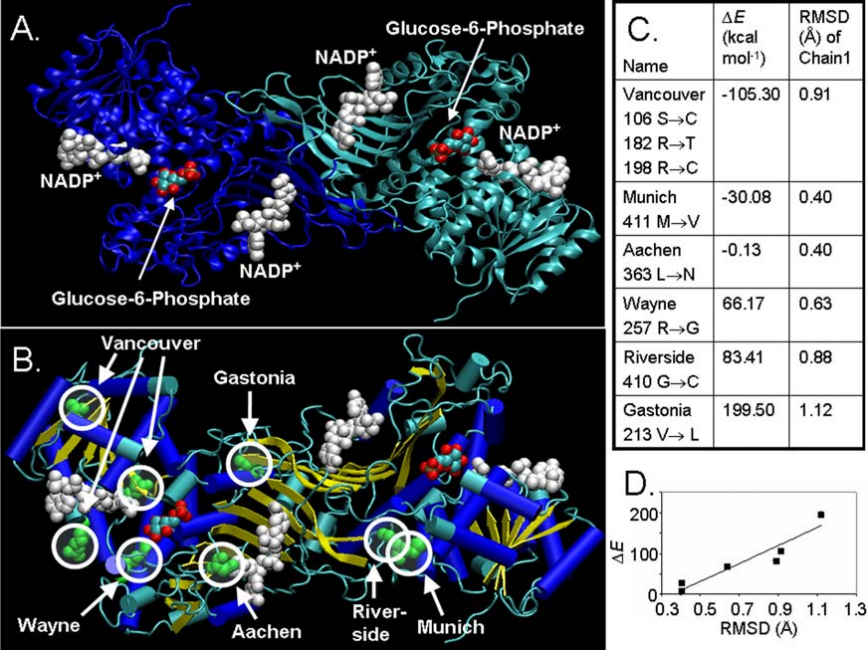
Molecular modeling of G6PD variants of German ancestry. (A) Wild-type structure of G6PD, with NADP^+^ entries from 2BH9 and G6P entries from 2BHL (shown as van der Waals representation). The two chains are shown in green and blue. (B) The locations of the six mutations is shown as green Van der Waal radii, highlighted by red circles. Beta sheets are shown in yellow color, while alpha helices are shown in blue color. (C and D) The root mean square is taken over one monomer, comparing the optimized structure of the mutant and that of the wild type. Figures were created with VMD software [28].

On the basis of relocation of all entries of two monomers of 2BH9 over those of 1QKI, the resulting structure is shown in Figure 1A. Each G6PD monomer has two NADP^+^ units and one G6P unit. All six mutations coming from German ancestry are highlighted in Van der Waals presentations in red circles in Figure 1B. Except for the Vancouver mutation, which is a triple mutant, all other mutations in German populations are single point mutations (Figure 1C). All mutations present in the German population are either close to one of the three G6P or NADP^+^ units or to the interface of the two monomers. Two of the three mutated amino acids, i.e. Arg198Cys and Trp182Trp are close to the binding site of NADP^+^. The Aachen mutation is close to the second NADP^+^ unit. The Wayne Arg198Gly mutation is close to the G6P binding region. Thus, these mutations may affect the binding of G6P and NADP^+^ units. Three mutations, i.e. Munich, Riverside and Gastonia, lie close to the interface of the two monomers. Thus these may affect the interaction between the two monomers. None of the mutations introduced a major structural change upon energy minimization. All mutations are within an RMSD value of 1.2 Å (Figure 1D), indicating that the mutations can be accommodated by simple flexible deformation of the protein.

Next, we compared the clinical appearance of these and other G6PD variants from Germany with those from other populations. In addition to the G6PD variants from German ancestry described in Figure 1, a number of other variants have been described in the past, though without knowledge of the exact mutation at the DNA level. The classification according to the clinical symptoms showed that most of these variants belong to the classes 1 or two (Table 1). Only one variant was a borderline class 2 or 3 G6PD deficiency (G6PD Frankfurt).

**Table 1:** Classification of G6PD variants from German ancestry

G6PD variant	Class	Reference
G6PD Aachen	1	[20]
G6PD Berlin	1	[29]
G6PD Bielefeld	2	[30]
G6PD Bodensee (Schwaben)	2	[31]
G6PD Cologne 1	2	[32]
G6PD Cologne 2	1	[32]
G6PD Cologne 3	2	[32]
G6PD Frankfurt	2 or 3	[33]
G6PD Freiburg	1	[34], [35]
G6PD Gastonia	1	[36]
G6PD Hamburg	1	[37]
G6PD Hamm	1	[38]
G6PD Iserlohn	2	[39]
G6PD Magdeburg	2	[40]
G6PD Moosburg	1	[41]
G6PD Munich	1	E. Beutler, personal communication
G6PD Regensburg	1	[39]
G6PD Riverside	1	[42]
G6PD Tübingen	1	[43], [44]
G6PD Vancouver	1	[45]
G6PD Wayne	1	[46]
G6PD Zähringen	2	[47]

## Discussion

G6PD deficiency is very frequent in Africa, Middle East, and Southeast Asia, but rare in Northern Europe (including Germany) or Northern America (except African Americans). This raises the question, whether differences in population genetics might account for this phenomenon. Rather than genetic or biological borders among human populations and tribes, the geographical distribution contributes to the cumulative occurrence of different forms of G6PD deficiency. The Mediterranean variants are not or rarely found in Asia or America. The fact that Japan is an island may explain the restricted occurrence of the G6PD Japan variant to this geographical area [12].

In the present investigation, we describe the three-dimensional localization of the structural modification in six G6PD class 1 variants of German origin. It is intriguing that none of the common Mediterranean variants are found in Germany as of yet. As can be seen in Table 2 and 3, some G6PD variants are restricted to certain areas and regions, while others are more widely spread. G6PD variants with locally restricted occurrence speak for an independent development during evolution. On the other side, a number of G6PD variants were found in Mediterranean and Middle-Eastern European populations as well as in the Near and Middle East suggesting a significant gene flow from Near East both to South-Eastern Europe and via sub-Saharan Africa to the South Mediterranean [13], [14]. An enhanced distribution of G6PD deficiency may have taken place by Greek settlers, who established many colonies throughout the Mediterranean world in ancient times [15]. In spite of this distribution of G6PD deficiency over Europe the question arises, why the common Mediterranean mutations were not found in German populations suffering from G6PD deficiency.

**Table 2:** Ubiquitous G6PD variants in Europe, Near East, and Africa

G6PD variant	Mutation	Countries and regions
G6PD A-	202 G → A plus 376 A → G	Near East, Poland, Italy, Spain, Nigeria, Mauritius,
G6PD Aures	143 T→C	Near East, Spain, Algeria
G6PD Cassano	1347 G→C	Greece, Croatia, Italy
G6PD Chatham	1003 G→A	Near East, Italy, Spain
G6PD Cosenza	1376 G → C	Croatia, Italy
G6PD Malaga	542 A→T	Poland, Spain
G6PD Mediterranean	563 C → T plus 1311 C → T	Near East, Greece, Croatia, Bulgaria, Italy, Spain, Mauritius
G6PD Santamaria	542 A→T plus 376 A→G	Italy, Spain, Algeria
G6PD Seattle	844 G→C	Greece, Croatia, Bulgaria, Italy, Spain, Algeria,
G6PD Tokyo	1246 G →A	Poland, Italy
G6PD Union	1360 G → A	Croatia, Italy, Spain

References: See supplementary file (Table S1)

**Table 3:** Endemic G6PD variants in Europe, Near East, and Africa

Country	G6PD variant
Africa: Mauritius	G6PD Orissa
Near East	G6PD-Mediterranean-like
Jewish ancestry	G6PD Rehovot; G6PD Meshadi
Greece	G6PD Ierapetra; G6PD Hermoupolis; G6PD Acrokorinthos
Croatia	G6PD Split
Bulgaria	G6PD Corinth; G6PD Ohut II; G6PD Rudosem; G6PD Nedelino;
	G6PD Kilgore; G6PD Boston; G6PD Poznan; G6PD Panay
Poland	G6PD Radlowo; G6PD Torun; G6PD Beverly Hills; G6PD Nashville; G6PD Puerto Limon
Czech Republic	G6PD Varnsdorf; G6PD Praha
Italy	G6PD Montalbano; G6PD S. Antioco; G6PD Cosenza; G6PD Partenope;
	G6PD Tokyo-like; G6PD Neapolis; G6PD Sao Borja; G6PD Cagliari; G6PD Sassari;
	G6PD Ferrara II; G6PD Modena; G6PD Lodi; G6PD Lagosanto; G6PD Coimbra;
	G6PD Sibari; G6PD Maewo
Spain	G6PD Tomah; G6PD Murcia; G6PD Valladolid; G6PD Madrid; G6PD Clinic

References: See supplementary file (Table S2)

Different hypotheses can be entertained to explain the differing occurrence of G6PD deficiency in various areas and populations. One point of view is that G6PD deficiency has independently developed with comparable frequencies in different areas. This is true for G6PD class 1 variants, which are found as sporadic cases worldwide and which cause chronic hemolytic anemia. Class 2 or class 3 G6PD deficiency is different from class 1. These forms are better tolerated except in case of oxidative stress leading to a hemolytic crisis. Class 2 and 3 variants have been selected by malaria. Diminished concentrations of reduced glutathione may represent a permissive environment for protozoal parasites such as *Plasmodium falciparum*. Hence, G6PD mutations are protective against malaria [16]. A comparable convergent evolution between protecting G6PD mutations and thalassemia or sickle cell anemia has been proposed [17], [18]. Malaria, thalassemia, and sickle cell anemia may, therefore, exert a positive selection pressure for carriers of G6PD mutations. As malaria is not endemic in Germany, but was present in certain Mediterranean (i.e. Sardinia), African and Arabian regions in former times, it is reasonable to speculate that for this reason G6PD deficiency class 3 is not as frequent as in areas plagued with malaria. Indeed, G6PD variants class 3 from Germany are rarely described. Except of G6PD Frankfurt, no other form appeared in the medical literature during the past six decades (Table 3). This is in contrast to the occurrence of G6PD variants on other populations, where class 3 mutants appear with a much higher frequency [19]

It could also be argued that the *Limes Germanicus* (Latin for German frontier) of the ancient Roman Empire, which separated the roman empire from un-subdued German tribes and had its maximum extent in the second century A.D., had a barrier function further restricting the distribution of G6PD deficiency in Middle European areas (if not distributed before the Roman Empire and the *Limes* were established). Class 1 variants are sporadic by definition, and apparently do not provide any advantage against malaria. *Falciparum* malaria, the selective force responsible for the expansion of class 2 G6PD variants (typically, G6PD Mediterranean), was never present in Northern Europe. The prevalence of *falciparum* (malignant) malaria was low during the Roman Empire period. After the fall of the Empire, *falciparum* malaria exploded and became endemic in some Southern region due to the collapse of the irrigation system and expansion of marshes. The Roman citizens (*cives romani*) were a minority in the Northern provinces and largely outnumbered by the Romanized local populations. Finally, the *Limes* was not an efficient barrier against the inflowing large Germanic populations (*barbari*) that came in during the following centuries. Very extensive admixture of different German populations occurred later on, i.e., during the Thirty Years' War (1618–1648) and recently after the huge population shifts in Germany after World War II. Hence, the hypothesis of a barrier function of the Roman limes is not convincing.

The fact that G6PD-deficiency is rare in Germany but relatively common in the Mediterranean region and Northern Africa gave rise to speculations in the past that G6PD-deficiency was spread into German populations, as the Roman Empire had occupied the countries beyond the Alps. Offspring of Roman soldiers of Mediterranean or Northern African origin and German inhabitants might have carried G6PD mutations leading to G6PD-deficiency in German populations nowadays. This opinion, which might have its roots in fascistic mindsets, seemed reasonable at superficial consideration in ages when solely clinical or biochemical data of G6PD deficiency were available. As of now, all G6PD mutations of German ancestries are different at the DNA level from the known Mediterranean and Northern African forms of G6PD-deficiency. This clearly disproves the ambiguous idea that G6PD deficiency appeared in Germany by mating of Roman soldiers and German women.

In areas where malaria does not represent a challenge for human populations, other weak influences may affect the manifestation of G6PD deficiency. A negative selection pressure has been discussed for non-lethal disorders or rare inherited syndromes. Decreased amounts of reduced glutathione may favour disorders such as manic depression, red/green colour vision, multiple sclerosis, diabetes mellitus, cataract, fragile X-syndrome and others. A relationship between lethal diseases such as cancer and G6PD deficiency has also been proposed. However, all these associations are weak. If any, these factors may have a weak influence on the manifestation of G6PD deficiency and may lead to a reduction of the frequency of G6PD deficiency in German populations. The most likely possibility is that the sporadic variants that are observed in Germany really represent the effect of random mutations. The mutations that are seen, then, represent the balance between the rate at which these mutations occur and the rate at which they are lost from the population. Except for the class 1 variants, which are probably lost very rapidly, most G6PD deficiency has very little effect on fitness and is therefore relatively slowly lost from the population.

## Materials and Methods

The German variants G6PD Aachen [20], [21] and Munich [7] and their mutations in the *G6PD* gene have been reported. G6PD Wayne [22], G6PD Vancouver [23], G6PD Gastonia, and G6PD Riverside (E. Beutler, unpublished data) are variants of patients of German emigrants.

Three crystal structures available at the Protein Data Bank (PDB, http://www.rcsb.org/pdb/) from human G6PD with PDB codes 1QKI [10], 2BH9 and 2BHL [11] are similar in their backbone folding. 1QKI (resolution = 3.00 Å) is an octamer consisting of four dimers, each with a point mutation at 459 Arg->Lys. 2BH9 (resolution = 2.50 Å) has been crystallized as a monomer with two NADP^+^ units [11], while 2BHL (resolution = 2.90 Å) has been crystallized as a dimer with two G6P units. Both structures are without any mutation, but 2BHL has 25 N-terminal residues missing. Since the smallest human glucose-6-phosphate dehydrogenase functional unit is a dimer [24], [25], we started with a dimeric structure (Figure 1A). The structure was obtained by positioning two 2BH9 monomers (along with their NADP^+^ units) as in the dimer of 1QKI. The G6P molecules were taken from 2BHL after it was overlapped onto 2BH9 monomers. Crystal water molecules were deleted. The mutant structures were generated in a four-step procedure. a) Side chain replacement according to mutant, b) energy minimization while keeping all atoms except the side chain fixed, c) energy minimization by allowing the side chain and all atoms around 8 Å of the side chain to move, and d) energy minimization while allowing all atoms to move. Energy minimization was done at each step to a final root-mean square gradient of 0.1 kcal/mol/Å using the MAB force field [26]. All mutants listed in Figure 1B were modelled and the resulting structures were overlapped over the Wild Type. All modelling was done using the Moloc program [27].

## Supporting Information

Table S1 Ubiquitous G6PD variants in Europe, Near East, and Africa
(0.06 MB DOC)

Table S2 Endemic G6PD variants in Europe, Near East, and Africa
(0.05 MB DOC)
