# Supplementary material for: Three-Dimensional Modeling of Glucose-6-phosphate Dehydrogenase-Deficient Variants from German Ancestry
Source: PLoS One. 2007 Jul 18;2(7):e625. doi: 10.1371/journal.pone.0000625 (PMC1913203; doi:10.1371/journal.pone.0000625)
Supplement: Table S1 — Ubiquitous G6PD variants in Europe, Near East, and Africa (0.06 MB DOC) [file pone.0000625.s001.doc]

**Faaroq Ahmad Kiani, Sonja Schwarzl, Stefan Fischer, and Thomas Efferth.**

**Three-dimensional modelling of glucose-6-phosphate dehydrogenase-deficient variants from German ancestry**

**Plos One**

**Supplementary File (Table S1)**

| **Table 2:** Ubiquitous G6PD variants in Europe, Near East, and Africa | | |
| --- | --- | --- |
|  |  |  |
| **G6PD variant** | **Mutation** | **Countries and regions** |
| G6PD Mediterranean | 563 C → T plus 1311 C → T | Near East, Greece, Croatia, Bulgaria, Italy, Spain, Mauritius |
| G6PD A- | 202 G → A plus 376 A → G | Near East, Poland, Italy,Spain, Nigeria, Mauritius, |
| G6PD Seattle |  | Greece, Croatia, Bulgaria, Italy, Spain, Algeria, |
| G6PD Aures |  | Near East, Spain, Algeria |
| G6PD Union | 1360 G → A | Croatia, Italy,Spain |
| G6PD Santamaria |  | Italy, Spain, Algeria |
| G6PD Cassano |  | Greece, Croatia, Italy |
| G6PD Chatham |  | Near East, Italy, Spain |
| G6PD Malaga |  | Poland, Spain |
| G6PD Tokyo | 1246 G →A | Poland, Italy |
| G6PD Cosenza | 1376 G → C | Croatia, Italy |

| **G6PD variant** | **Reference** |  |
| --- | --- | --- |
| G6PD A- | Calabro et al., 1993; Ninfali et al., 1993; Alfinito et al., 1997; | |
|  | Citadella et al., 1997; Pietrapertosa et al., 2001; Jablonska-Skwiecinska et al., 1999 | |
|  | Vives et al., 1982; Cladera Serra et al., 1997 | |
| G6PD Aures | Vives et al., 1982; Cladera Serra et al., 1997 | |
| G6PD Cassano | Calabro et al., 1993; Alfinito et al., 1997; Menounos et al., 2000; Barisic et al., 2005 | |
| G6PD Chatham | Citadella et al., 1997; Cladera Serra et al., 1997 | |
| G6PD Cosenza | Calabro et al., 1993; Alfinito et al., 1997; Barisic et al., 2005 | |
| G6PD Malaga | Jablonska-Skwiecinska et al., 1999; Vulliamy et al., 1996; Cladera Serra et al., 1997 | |
| G6PD Mediterranean | Corcoran et al., 1992; Calabro et al., 1993; Frigerio et al., 1994; Cappellini et al., 1996; | |
|  | Alfinito et al., 1997; Citadella et al., 1997; Martinez et al., 1997; Menounos et al., 2000; | |
|  | Barisic et al., 2005; Terzic et al., 1995; Shatskaya et al., 1980; | |
|  | Vives et al., 1982; Cladera Serra et al., 1997; Vives-Corrons et al., 1990; | |
| G6PD Santamaria | Ninfali et al., 1993; Alfinito et al., 1997; Citadella et al., 1997; Vulliamy et al., 1996; | |
|  | Cladera Serra et al., 1997 |  |
| G6PD Seattle | Calabro et al., 1993; Cappellini et al., 1995; Pietrapertosa et al., 2001; Alfinito et al., 1997; | |
|  | Martinez et al., 1997; Menounos et al., 2000; Barisic et al., 2005; Toncheva and | |
|  | Tzoneva, 1984; Cladera Serra et al., 1997; | |
| G6PD Tokyo | Martinez et al., 1997; Jablonska-Skwiecinska et al., 1999 | |
| G6PD Union | Cappellini et al., 1996; Alfinito et al., 1997; Martinez et al., 1997; Barisic et al., 2005; | |

**References to Table 2:**

Alfinito F, Cimmino A, Ferraro F, Cubellis MV, Vitagliano L, Francese M, Zagari A, Rotoli B, Filosa S, Martini G. Molecular characterization of G6PD deficiency in Southern Italy: heterogeneity, correlation genotype-phenotype and description of a new variant (G6PD Neapolis). Br J Haematol 1997;98:41-6.

Barisic M, Korac J, Pavlinac I, Krzelj V, Marusic E, Vulliamy T, Terzic J. Characterization of G6PD deficiency in southern Croatia: description of a new variant, G6PD Split. J Hum Genet 2005;50:547-9.

Calabro V, Mason PJ, Filosa S, Civitelli D, Cittadella R, Tagarelli A, Martini G, Brancati C, Luzzatto L. Genetic heterogeneity of glucose-6-phosphate dehydrogenase deficiency revealed by single-strand conformation and sequence analysis. Am J Hum Genet 1993;52:527-36.

Calabro V, Mason PJ, Filosa S, Civitelli D, Cittadella R, Tagarelli A, Martini G, Brancati C, Luzzatto L. Genetic heterogeneity of glucose-6-phosphate dehydrogenase deficiency revealed by single-strand conformation and sequence analysis. Am J Hum Genet 1993;52:527-36.

Cappellini MD, Martinez di Montemuros F, De Bellis G, Debernardi S, Dotti C, Fiorelli G. Multiple G6PD mutations are associated with a clinical and biochemical phenotype similar to that of G6PD Mediterranean. Blood 1996;87:3953-8.

Cappellini MD, Martinez di Montemuros F, Dotti C, Tavazzi D, Fiorelli G. Molecular characterisation of the glucose-6-phosphate dehydrogenase (G6PD) Ferrara II variant. Hum Genet 1995;95:440-2.

Cittadella R, Civitelli D, Manna I, Azzia N, Di Cataldo A, Schiliro G, Brancati C. Genetic heterogeneity of glucose-6-phosphate dehydrogenase deficiency in south-east Sicily. Ann Hum Genet 1997;61:229-34.

Cladera Serra A, Oliva Berini E, Torrent Quetglas M, Bartolozzi Castilla E. [Prevalence of glucose-6-phosphate dehydrogenase deficiency in a student population on the island of Menorca] Article in spanish. Sangre (Barc) 1997;42:363-7.

Corcoran CM, Calabro V, Tamagnini G, Town M, Haidar B, Vulliamy TJ, Mason PJ, Luzzatto L. Molecular heterogeneity underlying the G6PD Mediterranean phenotype. Hum Genet 1992;88:688-90.

Frigerio R, Sole G, Lovicu M, Passiu G. Molecular and biochemical data on some glucose-6-phosphate dehydrogenase variants from southern Sardinia. Haematologica 1994;79:319-21.

Jablonska-Skwiecinska E, Lewandowska I, Plochocka D, Topczewski J, Zimowski JG, Klopocka J, Burzynska B. Several mutations including two novel mutations of the glucose-6-phosphate dehydrogenase gene in Polish G6PD deficient subjects with chronic nonspherocytic hemolytic anemia, acute hemolytic anemia, and favism. Hum Mutat 1999;14:477-84.

Martinez di Montemuros F, Dotti C, Tavazzi D, Fiorelli G, Cappellini MD. Molecular heterogeneity of glucose-6-phosphate dehydrogenase (G6PD) variants in Italy. Haematologica 1997;82:440-5.

Menounos P, Zervas C, Garinis G, Doukas C, Kolokithopoulos D, Tegos C, Patrinos GP. Molecular heterogeneity of the glucose-6-phosphate dehydrogenase deficiency in the Hellenic population. Hum Hered 2000;50:237-41. Erratum in: Hum Hered 2000;50:369.

Ninfali P, Bresolin N, Baronciani L, Fortunato F, Comi G, Magnani M, Scarlato G. Glucose-6-phosphate dehydrogenase Lodi844C: a study on its expression in blood cells and muscle. Enzyme 1991;45:180-7.

Pietrapertosa A, Palma A, Campanale D, Delios G, Vitucci A, Tannoia N. Genotype and phenotype correlation in glucose-6-phosphate dehydrogenase deficiency. Haematologica 2001;86:30-5.

Shatskaya TL, Krasnopolskaya KD, Tzoneva M, Mavrudieva M, Toncheva D. Variants of erythrocyte glucose-6-phosphate dehydrogenase (G6PD) in Bulgarian populations. Hum Genet 1980;54:115-7.

Terzic J, Krzelj V, Drmic I, Andelinovic S, Primorac D, Mestrovic J, Balarin L. Genetic analysis of the glucose-6-phosphate dehydrogenase deficiency in a southern Croatia. Coll Antropol 1998;22:485-9.

Toncheva D, Tzoneva M. Genetic polymorphism of G6PD in a Bulgarian population. Hum Genet 1984;67:340-2.

Vives Corrons JL, Pujades A. Heterogeneity of "Mediterranean type" glucose-6-phosphate dehydrogenase (G6PD) deficiency in Spain and description of two new variants associated with favism. Hum Genet 1982;60:216-21.

Vives-Corrons JL, Kuhl W, Pujades MA, Beutler E. Molecular genetics of the glucose-6-phosphate dehydrogenase (G6PD) Mediterranean variant and description of a new G6PD mutant, G6PD Andalus1361A. Am J Hum Genet 1990;47:575-9.

Vulliamy T, Rovira A, Yusoff N, Colomer D, Luzzatto L, Vives-Corrons JL. Independent origin of single and double mutations in the human glucose 6-phosphate dehydrogenase gene. Hum Mutat 1996;8:311-8.
