# Supplementary material for: Three-Dimensional Modeling of Glucose-6-phosphate Dehydrogenase-Deficient Variants from German Ancestry
Source: PLoS One. 2007 Jul 18;2(7):e625. doi: 10.1371/journal.pone.0000625 (PMC1913203; doi:10.1371/journal.pone.0000625)
Supplement: Table S2 — Endemic G6PD variants in Europe, Near East, and Africa (0.05 MB DOC) [file pone.0000625.s002.doc]

**Faaroq Ahmad Kiani, Sonja Schwarzl, Stefan Fischer, and Thomas Efferth.**

**Three-dimensional modelling of glucose-6-phosphate dehydrogenase-deficient variants from German ancestry**

**Plos One**

**Supplementary File (Table S2)**

| **Table 3:** Endemic G6PD variants in Europe, Near East, and Africa | |  |
| --- | --- | --- |
|  |  |  |
| **Country** | **G6PD variant** | **Reference** |
| **Africa: Mauritius** | G6PD Orissa | Kotea et al., 1999 |
| **Near East:** | G6PD-Mediterranean-like | el-Hazmi et al., 1994 |
| **Jewish ancestry:** | G6PD Rehovot; G6PD Meshadi | Ianovici-Kidon et al., 2000; Lim et al., 2005 |
| **Greece:** | G6PD Ierapetra; G6PD Hermoupolis; G6PD Acrokorinthos | Menounos et al., 2000 |
| **Croatia** | G6PD Split | Barisic et al., 2005 |
| **Bulgaria** | G6PD Corinth; G6PD Ohut II; G6PD Rudosem; G6PD Nedelino | Shatskaya et al., 1980; |
|  | G6PD Kilgore; G6PD Boston; G6PD Poznan; G6PD Panay | Toncheva and Tzoneva, 1984 |
| **Poland:** | G6PD Radlowo; G6PD Torun; G6PD Beverly Hills; G6PD Nashville; G6PD Puerto Limon | Jablonska-Skwiecinska et al., 1999 |
| **Czech Republic:** | G6PD Varnsdorf; G6PD Praha | Xu et al., 1995 |
| **Italy:** | G6PD Montalbano; G6PD S. Antioco; G6PD Cosenza; G6PD Partenope; | Fenu et al., 1982; Calabro et al., 1990; |
|  | G6PD Tokyo-like; G6PD Neapolis; G6PD Sao Borja; G6PD Cagliari; G6PD Sassari; | Corcoran et al., 1992; Ninfali et al., 1993; |
|  | G6PD Ferrara II; G6PD Modena; G6PD Lodi; G6PD Lagosanto; G6PD Coimbra; | Cappellini et al., 1996; Alfinito et al., 1997; |
|  | G6PD Sibari; G6PD Maewo | Citadella et al., 1997; Martinez et al., 1997 |
|  |  | Pietrapertosa et al., 2001 |
| **Spain:** | G6PD Tomah; G6PD Murcia; G6PD Valladolid; G6PD Madrid; G6PD Clinic | Vives et al., 1982; Cladera Serra et al., 1997; |
|  |  | Vives et al., 1982; |

**References to Table 3:**

Alfinito F, Cimmino A, Ferraro F, Cubellis MV, Vitagliano L, Francese M, Zagari A, Rotoli B, Filosa S, Martini G. Molecular characterization of G6PD deficiency in Southern Italy: heterogeneity, correlation genotype-phenotype and description of a new variant (G6PD Neapolis). Br J Haematol 1997;98:41-6.

Barisic M, Korac J, Pavlinac I, Krzelj V, Marusic E, Vulliamy T, Terzic J. Characterization of G6PD deficiency in southern Croatia: description of a new variant, G6PD Split. J Hum Genet 2005;50:547-9.

Calabro V, Giacobbe A, Vallone D, Montanaro V, Cascone A, Filosa S, Battistuzzi G. Genetic heterogeneity at the glucose-6-phosphate dehydrogenase locus in southern Italy: a study on a population from the Matera district. Hum Genet 1990;86:49-53.

Cappellini MD, Martinez di Montemuros F, De Bellis G, Debernardi S, Dotti C, Fiorelli G. Multiple G6PD mutations are associated with a clinical and biochemical phenotype similar to that of G6PD Mediterranean. Blood 1996;87:3953-8.

Cittadella R, Civitelli D, Manna I, Azzia N, Di Cataldo A, Schiliro G, Brancati C. Genetic heterogeneity of glucose-6-phosphate dehydrogenase deficiency in south-east Sicily. Ann Hum Genet 1997;61:229-34.

Cladera Serra A, Oliva Berini E, Torrent Quetglas M, Bartolozzi Castilla E. [Prevalence of glucose-6-phosphate dehydrogenase deficiency in a student population on the island of Menorca] Article in spanish. Sangre (Barc) 1997;42:363-7.

Corcoran CM, Calabro V, Tamagnini G, Town M, Haidar B, Vulliamy TJ, Mason PJ, Luzzatto L. Molecular heterogeneity underlying the G6PD Mediterranean phenotype. Hum Genet 1992;88:688-90.

El-Hazmi MA, Jabbar FA, Al-Faleh FZ, Al-Swailem AR, Warsy AS. Patterns of sickle cell, thalassaemia and glucose-6-phosphate dehydrogenase deficiency genes in north-western Saudi Arabia. Hum Hered 1991;41:26-34.

Fenu MP, Finazzi G, Manoussakis C, Palomba V, Fiorelli G. Glucose-6-phosphate dehydrogenase deficiency: genetic heterogeneity in Sardinia. Ann Hum Genet 1982; 46:105-114.

Iancovici-Kidon M, Sthoeger D, Abrahamov A, Wolach B, Beutler E, Gelbart T, Barak Y. A new exon 9 glucose-6-phosphate dehydrogenase mutation (G6PD "Rehovot") in a Jewish Ethiopian family with variable phenotypes. Blood Cells Mol Dis 2000;26:567-71. Erratum in: Blood Cells Mol Dis 2001;27:351. Volach B [corrected to Wolach B].

Jablonska-Skwiecinska E, Lewandowska I, Plochocka D, Topczewski J, Zimowski JG, Klopocka J, Burzynska B. Several mutations including two novel mutations of the glucose-6-phosphate dehydrogenase gene in Polish G6PD deficient subjects with chronic nonspherocytic hemolytic anemia, acute hemolytic anemia, and favism. Hum Mutat 1999;14:477-84.

Kotea R, Kaeda JS, Yan SL, Sem Fa N, Beesoon S, Jankee S, Ramasawmy R, Vulliamy T, Bradnock RW, Bautista J, Luzzatto L, Krishnamoorthy R, Mason PJ. Three major G6PD-deficient polymorphic variants identified among the Mauritian population. Br J Haematol 1999;104:849-54.

Lim F, Vulliamy T, Abdalla SH. An Ashkenazi Jewish woman presenting with favism. J Clin Pathol 2005;58:317-9.

Martinez di Montemuros F, Dotti C, Tavazzi D, Fiorelli G, Cappellini MD. Molecular heterogeneity of glucose-6-phosphate dehydrogenase (G6PD) variants in Italy. Haematologica 1997;82:440-5.

Menounos P, Zervas C, Garinis G, Doukas C, Kolokithopoulos D, Tegos C, Patrinos GP. Molecular heterogeneity of the glucose-6-phosphate dehydrogenase deficiency in the Hellenic population. Hum Hered 2000;50:237-41. Erratum in: Hum Hered 2000;50:369.

Ninfali P, Baronciani L, Ruzzo A, Fortini C, Amadori E, Dall'ara G, Magnani M, Beutler E. Molecular analysis of G6PD variants in northern Italy: a study on the population from the Ferrara district. Hum Genet. 1993 Sep;92(2):139-42.

Pietrapertosa A, Palma A, Campanale D, Delios G, Vitucci A, Tannoia N. Genotype and phenotype correlation in glucose-6-phosphate dehydrogenase deficiency. Haematologica 2001;86:30-5.

Shatskaya TL, Krasnopolskaya KD, Tzoneva M, Mavrudieva M, Toncheva D. Variants of erythrocyte glucose-6-phosphate dehydrogenase (G6PD) in Bulgarian populations. Hum Genet 1980;54:115-7.

Toncheva D, Tzoneva M. Genetic polymorphism of G6PD in a Bulgarian population. Hum Genet 1984;67:340-2.

Vives Corrons JL, Pujades A. Heterogeneity of "Mediterranean type" glucose-6-phosphate dehydrogenase (G6PD) deficiency in Spain and description of two new variants associated with favism. Hum Genet 1982;60:216-21.

Xu W, Westwood B, Bartsocas CS, Malcorra-Azpiazu JJ, Indrak K, Beutler E. Glucose-6 phosphate dehydrogenase mutations and haplotypes in various ethnic groups. Blood 1995;85:257-63.
